# 

**Published:** 1888-04

**Authors:** 


					﻿io cts. a copy
APRIL, 1883.
$1.00 per year.
HALES
eJoVRNALJ
HEALTH
ESTABLISHED 18J)4
IK. 35
No. 4.
OFFICE?. 206 BROADWAY, EVENING POST BUILDING? Room 11. N.
Entered as Second-class Matter at the Post-Office, New York.
				

